# Health sciences library workshops in the COVID era: librarian perceptions and decision making

**DOI:** 10.5195/jmla.2023.1663

**Published:** 2023-07-10

**Authors:** Nell Aronoff, Molly K. Maloney, Amy G. Lyons, Elizabeth Stellrecht

**Affiliations:** 1 naronoff@buffalo.edu, Associate Librarian, University Libraries, University at Buffalo, Buffalo, NY.; 2 mkm9@buffalo.edu, Senior Assistant Librarian, University Libraries, University at Buffalo, Buffalo, NY.; 3 alyons@buffalo.edu, Librarian, University Libraries, University at Buffalo, Buffalo, NY.; 4 thomann@buffalo.edu, Librarian, University Libraries, University at Buffalo, Buffalo, NY.

**Keywords:** Workshops, COVID-19, surveys and questionnaires

## Abstract

**Objective:**

We sought to determine how the COVID-19 pandemic impacted academic health sciences library workshops. We hypothesized that health sciences libraries moved workshops online during the height of the pandemic and that they continued to offer workshops virtually after restrictions were eased. Additionally, we believed that attendance increased.

**Methods:**

In March 2022, we invited 161 Association of American Health Sciences Libraries members in the US and Canada to participate in a Qualtrics survey about live workshops. Live workshops were defined as synchronous; voluntary; offered to anyone regardless of school affiliation; and not credit-bearing. Three time periods were compared, and a chi square test of association was conducted to evaluate the relationship between time period and workshop format.

**Results:**

Seventy-two of 81 respondents offered live workshops. A chi square test of association indicated a significant association between time period and primary delivery method, chi-square (4, N=206) = 136.55, p< .005. Before March 2020, 77% of respondents taught in person. During the height of the pandemic, 91% taught online and 60% noted higher attendance compared to pre-pandemic numbers. During the second half of 2021, 65% of workshops were taught online and 43% of respondents felt that attendance was higher than it was pre-pandemic. Overall workshop satisfaction was unchanged (54%) or improved (44%).

**Conclusion:**

Most health sciences librarians began offering online workshops following the onset of the COVID-19 pandemic. More than half of respondents were still teaching online in the second half of 2021. Some respondents reported increased attendance with similar levels of satisfaction.

## INTRODUCTION

Health sciences librarians are important partners in education within academic settings. A recent scoping review revealed that instruction, reference, and medical education is the second most frequently cited category or role for health information professionals [1]. Librarians provide education in a variety of different ways, including by creating and offering library workshops. These workshops cover a range of topics such as citation management, database searching, scholarly communications issues, and data management; these offerings are often in response to an expressed need from library constituents [2–4].

Attendance at library workshops is often low. Workshop participation is voluntary and there is no cost to attend. Despite their good intentions, registrants' busy schedules can interfere with attendance and other commitments may take priority. It is also hard to know when the best times are to offer workshops. As a result, libraries have tried either to create new offerings, revamp current offerings, or solicit input for improvements to increase attendance of faculty, staff, and students [5–9].

Health sciences libraries have made workshops available virtually to lower possible barriers that may prevent participation [6, 10–12]. While online offerings are not new, the COVID-19 pandemic created an environment where virtual instruction was the only available avenue for educational programming. Librarians had to quickly learn how to provide content that they may have previously offered only in the physical classroom, including deciding whether they could or should move this content online [13–16].

The University at Buffalo is an R1 institution which serves five health sciences schools: Dental Medicine, Medicine and Biomedical Sciences, Nursing, Pharmacy and Pharmaceutical Sciences, and Public Health and Health Professions. In response to the COVID-19 pandemic, the entire institution shifted to virtual-only environment on March 16, 2020. When it became apparent that a return to in-person education was not going to occur in the coming weeks, the librarians on the Health Sciences Library Services (HSLS) team shifted to offering synchronous workshops via the Zoom platform. The HSLS team noticed both registration and attendance of library workshops increased during this time and made the decision to continue to offer workshops primarily online moving forward. However, once the institution returned to overall in-person education in the fall of 2021, workshop registration and attendance numbers dropped closer to pre-pandemic levels.

We wondered if other health sciences librarians observed similar attendance patterns. Our study sought to determine how the COVID-19 pandemic impacted academic health sciences library workshop offerings. We were particularly interested in data related to live workshops, which we defined as synchronous, voluntary, offered to anyone regardless of school affiliation, non-credit bearing and taught by a health sciences librarian. Specifically, we hypothesized that health sciences libraries across the country moved their workshops online during the height of the pandemic, and that the online availability of the workshops during this time led to an overall increase in attendance.

## METHODS

### Survey Development

An online survey instrument was developed using Qualtrics software that featured branching logic. All respondents answered the first two questions. To ensure that no duplicate responses were analyzed, respondents were asked to enter their institution name. The survey asked whether respondents' institutions offered live workshops. If a respondent's institution did not offer live workshops, branching logic directed the respondent to answer why not. After completing this question, these respondents were directed to the end of the survey. If a respondent reported that their institution offered live workshops, branching logic directed them to continue with the full survey that included 17 additional questions.

Our primary focus was to determine how the pandemic changed workshop format, attendance, and participant satisfaction. We also asked librarians to describe some of the successes and challenges they experienced teaching online workshops. The number of librarians who teach within the institution, the number of classes taught, and the breadth of topics presented were also questions of interest (see Appendix for complete list of questionnaire items).

Survey time periods were divided into three segments. The time prior to March 2020 was pre-pandemic. March 2020-June 2021 was called the height of the pandemic. We established July-December 2021 as a third time series, as at that point COVID-19 vaccines were more readily available and many universities saw more members of their communities returning to the libraries and campuses.

### Participants

We generated a list of potential survey participants by using the Association of Academic Health Sciences Libraries (AAHSL) website of Member Institutions. AAHSL was selected as it is the most comprehensive list of academic health sciences libraries in the United States and Canada and has been used in other studies similar in nature to our own. The target audience for the survey was health sciences librarians in the US and Canada that supported undergraduate, graduate, or professional programs. AAHSL members like the American Dental Association and the National Institutes of Health were excluded since they did not fit the inclusion criteria.

Using the AAHSL website, we built an Excel spreadsheet with column headers for the university name, library name, include, exclude, website, first name of contact, last name of contact, contact's email, and notes. This allowed us to perform a mail merge when distributing the survey via Outlook.

### Survey Distribution

Prior to distribution, the study protocol was submitted to the University's Institutional Review Board. It underwent Non-Committee Review and was determined to be exempt according to 45 CFR Part 46.104.

One hundred sixty-one invitations were emailed to the director or primary AAHSL contact on March 8, 2022, describing the study and requesting them to respond. Subjects were informed that the survey would take 10-15 minutes to complete. Respondents to the survey were not offered any tangible incentives to participate or complete the survey and were told that participation was voluntary.

Instructions for completing the survey stated that the director or primary contact could complete the survey. Alternatively, the director or contact could ask a colleague more familiar with workshop activities to respond. Instructions specified that only one person from the institution should complete the Qualtrics survey. Having only one person complete the survey was a way to ensure that multiple responses were not being returned from the same organization.

A first reminder email went out on March 15, 2022, and a final reminder was shared on March 22, 2022. The survey closed on March 23, 2022. Of the initial 161 survey invitations sent out, we received 81 final responses. Two “test” responses and 3 duplicate entries from the same institutions were removed prior to analysis.

### Data Analysis

A Qualtrics report was run on March 24, 2022. For each multiple-choice question, Qualtrics provided the minimum, maximum, mean, standard deviation, variance, count, and a bar graph. Questions that allowed respondents to select all that apply showed the percent that choice was selected, the count, and a bar graph. We reviewed “other” and open-ended responses and grouped them thematically.

## RESULTS

### Response Rate and Live Workshops in Health Sciences Libraries

The survey received eighty-one unique responses, a response rate of 50%, for the first question regarding whether their libraries offer live workshops. Of these, seventy-two (89%) offer live workshops. Two of the respondents did not complete the full survey, but the responses they supplied are included in the analysis.

The nine respondents (11%) who indicated their libraries do not offer live workshops were asked for the reasons behind this decision. They were given three choices and an “other” write-in option and could select all that apply. Of the provided responses, the most frequently cited were a lack of staffing or resources (n=4, 44%), low past attendance at workshops (n=4, 44%) and a lack of time (n=3, 33%). Five write-in responses included embedded or curricula-based instruction through a liaison model and health sciences offerings for affiliated audiences exclusively. One respondent indicated that no live workshops were offered due to COVID restrictions.

### Institution Characteristics

The 72 respondents that offer live workshops provided details about their institutions, the number of health sciences librarians responsible for workshop instruction, and the schools or programs they served. Institution size was based on the Carnegie Classifications [17]. Approximately 58% of respondents (n=42) work at medium or large four-year institutions, 26% (n=19) at institutions with exclusively graduate or professional students, and 13% (n=9) at very small or small four-year institutions. Two respondents (3%) were not sure of their institution's classification. Respondents were asked “How many health sciences librarians generally teach workshops at your institution?” Librarian instruction teams ranged in size from 1-2 (n=10, 14%), 3-4 (n=26, 36%), 5-6 (n=12, 17%), 7-8 (n=17, 24%), 9-10 (n=5, 7%), or greater than 10 (n=2, 3%).

When asked about the health sciences schools or programs their libraries support, respondents were given a selection of ten choices followed by the option to select “other” and write-in their own responses. Of the provided ten choices, Medicine (n=71, 99%), Biomedical Sciences (n=62, 86%), Nursing (n=49, 68%), Public Health (n=44, 61%), and Physical Therapy (n=41, 57%) were the best represented programs or schools served, followed by Occupational Therapy (n=34, 47%), Dental Medicine (n=29, 40%), Pharmacy (n=26, 36%), Social Work (n=16, 22%), and Veterinary Medicine (n=4, 6%). Physician Assistant, Optometry, Speech Language Pathology, and Medical Laboratory Sciences programs were the top write-in responses.

### Workshop Offerings

Respondents were asked to identify the kinds of workshops typically provided by their libraries. Thirteen options were provided followed by an “other” write-in option and they were asked to select all that apply. At this point, one person abandoned the survey, meaning there were 71 responses. The top responses were citation management (n=65, 92%), database specific training (n=62, 87%), basic library overview (n=58, 82%), systematic or scoping reviews (n=51, 72%), and publishing, including open access (n=44, 62%). Of the write-in responses (n=21, 30%), Evidence-based practice or medicine, literature searching (basic through advanced), health literacy, poster design, and writing (literature reviews and abstracts) were mentioned.

Respondents were asked how they and their colleagues make decisions about what to offer. They were given five options and a write-in option and could select all that applied. Faculty request (n=53, 75%) and continuing with what has been popular in the past (n=52, 73%) were the most selected responses, followed closely by trends in reference inquiries (n=49, 69%), student requests (n=43, 61%) and trends in medical librarianship (n=40, 56%). The most frequent responses from those who selected “other” (n=17, 24%) included the curriculum, further emphasis on user needs, as well as librarian capacity, expertise, and interest.

When asked how many total workshops their libraries typically offer per semester, respondents answered in ranges between 1-3 (n=14, 20%), 4-6 (n=10, 14%), 7-9 (n=13, 18%), 10-12 (n=9, 13%), or more than 13 (n=25, 35%).

### COVID and Workshop Delivery

Respondents were asked “When did your campus return to a fully in-person environment?” Of the 70 responses, 3% (n=2) indicated they returned to campus in Summer 2020, 11% (n=8) in Fall 2020, 4% (n=3) in Spring 2021, 16% (n=11) in Summer 2021, and 24% (n=17) in Fall 2021. Only 1% of respondents (n=1) at the time of the survey indicated their campus was still remote. Never remote received zero responses. The “other” option was selected by 40% of respondents (n=28). Sixteen respondents indicated they were hybrid, and others explained that the situation depended on class size, department, or COVID-19 variants.

**Figure 1:**
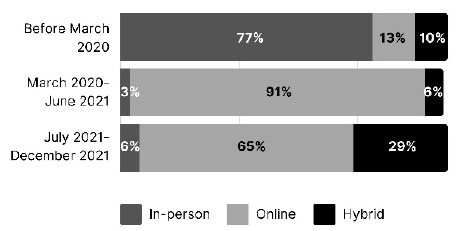
Primary delivery methods for health sciences library workshops across the three periods

Respondents were asked what the primary method of workshop delivery was during three time frames: before March 2020, March 2020-June 2021, and July 2021-December 2021. This included a definition of hybrid as “offering attendees the choice of attending the same live workshop in-person or online.” Results are reported in Figure 1.

A chi square test of association was conducted to evaluate the relationship between time period and primary method of delivery, using a significance level of .05. Results indicated a significant association between the two variables, chi-square (4, N=206) = 136.55, p< .005. Z tests using the Bonferroni correction indicated a significantly higher percentage of sessions given in-person before March 2020 compared to the other two time periods. In addition, the percentage of sessions given primarily in hybrid format was significantly higher for the July-December 2021 period than for either before March 2020 or between March 2020 and June 2021. Significant differences were found among all time periods for online training, with the highest percentage for the March 2020-June 2021 period, followed by July-December 2021, and the lowest percentage before March 2020.

Focusing on how librarians modified workshops because of the pandemic, respondents were given thirteen responses with the additional write-in option and were invited to select all that apply. Top selections included a change in mode of delivery (n=61/70, 87%), the addition of new workshops (n=44, 63%), and incorporating new accessibility features (n=43, 61%) or engagement features (n=38, 54%). Another change was that 39% of respondents (n=27) team taught workshops or had more than one librarian present. One write-in response stated offerings were stopped in part due to online fatigue from students. In addition, two write-in responses noted that they were providing workshops asynchronously. Respondents were also asked if there were any workshops that they were not able to offer after March 2020. Community based workshops and workshops that required special software or equipment like 3D printers were impacted by the pandemic.

### Attendance and Satisfaction

**Figure 2:**
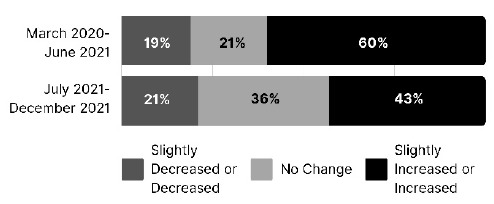
Changes in attendance at health sciences library workshops during two periods of the pandemic compared to pre-pandemic levels

To better understand librarian perceptions of COVID's impact on workshop attendance and satisfaction, respondents were asked to compare pre-pandemic attendance levels with the two subsequent periods. As the results in Figure 2 show, 60% of respondents saw attendance rise during the height of the pandemic and 43% felt attendance was higher during the second half of 2021 than it was pre-pandemic.

A chi square test of association indicated no statistically significant association between time period and perceived change in attendance (compared to pre-pandemic), chi-square (4, N=206) = 6.24, p = .18.

**Figure 3:**
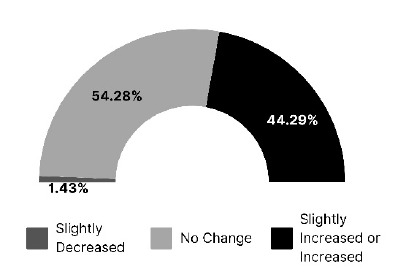
Librarian perception of overall satisfaction levels with health sciences library workshops after March 2020

Survey takers were asked about their perceived change in overall workshop satisfaction after March 2020. As Figure 3 indicates, 54% perceived no change, while 44% saw a slight increase or an increase. Only 1% noticed a slight decrease. No respondents perceived decreased satisfaction.

An open text response was provided for respondents to indicate the basis of their perception. Evaluations or surveys and participant feedback were widely used, while the format of the workshop and attendance served as a barometer for others. Seven respondents wrote that since they did not collect data, they could not address this question.

### Challenges, Successes, and the Future

Respondents were asked to select all that apply from seven provided challenges with an additional write-in option. The top responses included not being able to visually assess understanding (n=46/70, 66%), the technology learning curve for participants (n=35, 50%) and instructors (n=32, 46%), and hardware or software malfunctions (n=33, 47%).

With challenges come successes: respondents were asked to indicate what successes they and their colleagues experienced if they taught online from a list of four choices plus a write-in option. The majority agreed that new audiences could be reached as attendance was not tied to a physical location (n=64/70, 91%), followed by increased participation (n=43, 61%).

Moving forward, respondents are planning to offer workshops in a variety of formats: online and hybrid (n=1/70, 1%), in-person only (n=1, 1%), online only (n=3, 4%), hybrid only (n=14, 20%), in-person and online (n=24, 34%), and lastly the majority offering a combination of all three (n=27, 39%). No one indicated plans to discontinue workshops.

The final question was open-ended and asked respondents to share observations regarding how the pandemic affected workshop offerings, attendance, and future plans. Several respondents expressed frustration with hybrid workshops, with one person noting that hybrid workshops have “all the problems of an online platform and a face-to-face platform multiplied.” Some libraries recorded their workshops or provided access to asynchronous content. At one institution, participants watch a recording before coming to a half-hour-long Q&A session with a librarian.

## DISCUSSION

We sought to determine how COVID impacted live, standalone health sciences library workshops during the pandemic. We did not study educational efforts that were tied to course credit, workshops that were taught by outside instructors, or asynchronous or recorded offerings.

Our paper considers this one aspect of instruction. In their spring 2021 survey of academic library instruction during the pandemic, Shin et al. found that 69% of respondents gave online workshops during COVID and 64% planned to continue offering virtual workshops once they returned to campus [16].

Although responses from 81 AAHSL member institutions were received, this accounts for only half of the potential pool. Therefore, we do not know how other institutions' workshops were impacted by the pandemic. Our results demonstrate that close to 90% of respondents offer live library workshops and nearly half of respondents offer ten or more workshops each semester with topics ranging from basic library overviews to data management. More than 80% of respondents offer citation management, database-specific training, and basic library overview workshops.

We continue to offer workshops at the University at Buffalo, although we have had discussions about the value of workshops and whether there is enough return on investment to justify continuing to offer them. A little more than 10% of respondents indicated that they no longer offer workshops. Their reasons varied, but they were situational depending on the demands on their time and the needs of their organizations.

We hypothesized that health sciences libraries moved their workshops online during the height of the pandemic and anticipated that they continued to offer workshops virtually in the second half of 2021. The results largely confirmed our hypothesis. Before the pandemic, more than three quarters of respondents' primary method of workshop delivery was in-person. A global pandemic necessitated a format change, and between March 2020 and June 2021, 91% of respondents' primary mode was virtual. From July 2021-December 2021, as COVID restrictions were eased and vaccines became more readily available, nearly two-thirds of respondents were still offering workshops primarily online.

One of the main ways respondents modified workshops because of the pandemic was changing the mode of delivery. With this shift, instructors incorporated new accessibility and engagement features into their workshops. For example, software like Zoom offers live captioning, polling, and breakout rooms. Another pandemic-related adjustment made by more than a third of respondents was that workshops were team-taught or there was more than one librarian present.

Along those same lines, when asked about the challenges librarians experienced with online workshops, 29% of survey takers expressed a need for additional support. The most frequently cited challenge was that instructors found it hard to visually assess understanding in an online environment. An article written by a professor at Northern Arizona University one year into the pandemic noted that “it is unnerving to teach to a seemingly endless void of unresponsive black boxes” [18]. Similarly, a professor at the University of North Carolina at Wilmington found that “he can't rely on students' body language or the feeling in the room to gauge their understanding” [19]. In their 2021 study, Castelli and Sarvary discussed the benefits of having a camera on [20]. They wrote that “perhaps the most obvious benefit is the ability to communicate with nonverbal clues.” These clues help instructors “evaluate their teaching in real time and adjust accordingly.”

We asked respondents to gauge whether attendance and satisfaction were affected by the pandemic. We hypothesized that attendance increased during the pandemic, a trend reported by librarians at the University of Tennessee Health Science Center Health Sciences Library [21], Stony Brook Health Sciences Library [15], Stanford University's Lane Medical Library [22], NYU Health Sciences Library [23], and those surveyed by Shin et al. [16].

Compared to before March 2020, 60% of respondents saw an increase or a slight increase in attendance from March 2020-June 2021 and 42% saw an increase or slight increase from July 2021-December 2021. Thus, the increases were more pronounced during the height of the pandemic. In that same vein, 91% of survey takers agreed that teaching online enabled new audiences to participate in their workshops. The convenience of not having to travel to a physical location allowed health sciences libraries to reach more and different people. We speculate that as the pandemic continued, people grew weary of spending more time in virtual meetings. LaPolla, Contaxis, and Surkis also noticed a decline in attendance for their data sciences training series and posited that many professionals were experiencing “Zoom fatigue” that limited their interest in pursuing professional development opportunities [24].

Respondents reported that user satisfaction with their workshop programming was the same, if not better, with 54% of respondents observing no change and 44% of respondents observing slightly increased or increased satisfaction. This means that despite transitioning to a new format, perceived satisfaction stayed the same or improved. Given the nature of the survey and the way these questions were framed, respondents answered based on their perceptions. Even though many respondents were influenced by evaluations and surveys, perception is a subjective measure and may not represent the actual attendance or actual satisfaction.

Evaluating the success of workshops goes beyond attendance and satisfaction. Another challenge for health sciences librarians teaching standalone workshops is to understand the impact that these workshops have on research, teaching, learning, and patient care. At our institution, we request that workshop attendees complete a survey following the workshop. One question asks how attendees expect to apply what they learned, but there is no follow-up afterwards to better determine whether their anticipated use differed from their real use.

LaPolla, Contaxis, and Surkis studied longer-term impacts of the data workshops they offered at NYU [9] and Deardorff interviewed 14 researchers at the University of California, San Francisco before and three months after they completed introductory programming workshops [25]. Despite the low survey response rate, LaPolla, Contaxis, and Surkis's pilot confirmed that respondents benefitted from their workshops by building data collection and analysis skills. The pilot also helped inform the way they plan to collect data in the future (survey attendees only within a year of the workshop date). Deardoff concluded “that several participants made small changes to their workflows” but “reproducible behaviors did not change in any statistically significant manner.” These two studies demonstrate that workshops have an impact, but more research is needed on this topic.

In addition to exploring how standalone workshops benefit attendees, the benefits for librarians and libraries in offering these workshops also remains unclear. Given their well-established challenges and limitations, why do health sciences libraries continue to offer standalone workshops? Workshops may lead to research collaborations, more course-based instruction, or an increase in reference or research consultations, but a systematic investigation would deepen our understanding.

## Data Availability

Survey data associated with this article are available in the University at Buffalo Institutional Repository (UBIR) at http://hdl.handle.net/10477/84394.

## SUPPLEMENTAL FILES

**Appendix A:** Workshop Survey
