# Supplementary material for: Health sciences library workshops in the COVID era: librarian perceptions and decision making
Source: J Med Libr Assoc. 2023 Jul 10;111(3):657–64. doi: 10.5195/jmla.2023.1663 (PMC10361557; doi:10.5195/jmla.2023.1663)
Supplement: Supplementary file 1 — Appendix A: Workshop Survey [file jmla-111-3-657-s01.pdf]

Appendix A: Workshop Survey

1. Thank you for agreeing to participate in this survey. The objective of this study is to determine how COVID-19 has impacted academic health sciences library workshops. Participation in this study is voluntary and should take approximately 10 to 15 minutes to complete. You can discontinue the survey at any time.

2. Institution Name

3. Does your library offer live workshops? We define live workshops as: synchronous, voluntary, offered to anyone regardless of school affiliation, not credit-bearing, and taught by a health sciences librarian.
   - Yes
   - No

4. (If they select no in the third question) Why doesn't your library offer live workshops? Please select all that apply.
   - Do not have staffing or resources
   - Do not have time
   - Low attendance when offered in the past
   - Other, please specify below

   We thank you for your time spent taking this survey.
   Your response has been recorded.

5. (If they select yes in the third question) What is the size of your institution? Sizes are based on the Carnegie Classifications.
   - Very small four-year– FTE enrollment of fewer than 1,000 degree-seeking students
   - Small four-year – FTE enrollment of 1,000-2,999 degree-seeking students
   - Medium four-year – FTE enrollment of 3,000-9,999 degree-seeking students
   - Large four-year – FTE enrollment of at least 10,000 degree-seeking students
   - Exclusively graduate/professional with no undergraduate students
   - I do not know

6. How many health sciences librarians generally teach workshops at your institution?
   - 1-2
   - 3-4

- 5-6
- 7-8
- 9-10
- More than 10

7. What schools/programs does your library support? Please select all that apply.
    - Biomedical Sciences
    - Dental Medicine
    - Medicine
    - Nursing
    - Occupational Therapy
    - Pharmacy
    - Physical Therapy
    - Public Health
    - Social Work
    - Veterinary Medicine
    - Other, please specify below

8. What kinds of workshops does your library typically offer? Please select all that apply.
    - Basic Library Overview
    - Bioinformatics
    - Citation Management
    - Copyright
    - Critical Appraisal
    - Data Management or Analysis
    - Database Specific Training
    - Health Statistics
    - History of Medicine/Archives
    - Author Profiles (for example, ORCID)
    - Publishing, including Open Access Publishing
    - Research or Tenure Metrics
    - Systematic or Scoping Reviews
    - Other, please specify below

9. How do you and your colleagues decide what to offer? Please select all that apply.
    - Stick with what has been popular in the past
    - Faculty request
    - Student request
    - Notice similar reference inquiries
    - Trending topic in medical librarianship

- Other, please specify below

10. How many total workshops does your library typically offer each semester?
    - 1-3
    - 4-6
    - 7-9
    - 10-12
    - More than 13

11. When did your campus return to a fully in-person environment?
    - Summer 2020
    - Fall 2020
    - Spring 2021
    - Summer 2021
    - Fall 2021
    - Not yet. Everything is still remote.
    - Not applicable. Everything has been in-person.
    - Other, please specify below

12. What was the primary method of workshop delivery? We define hybrid as offering attendees the choice of attending the same live workshop in-person or online.

	In-person	Online	Hybrid	Other, please specify
Before March 2020				
March 2020-June 2021				
July 2021-December 2021				

13. How did you and your colleagues modify workshops as a result of the pandemic? Please select all that apply.
    - Did not adjust workshops
    - Changed mode of delivery
    - Discontinued certain workshops
    - Expand on existing workshops
    - Added new workshops
    - Adjusted the length of workshops
    - Adjusted the frequency of workshops

- Changed the presentation or workshop materials
- Incorporated new accessibility features (e.g. captioning, recording, transcripts)
- Incorporated different engagement features (e.g. polling, breakout rooms)
- Team teach or have more than one librarian present
- Hire or enlist new instructors
- Simplify material covered within a single workshop
- Other, please specify below

14. Compared with attendance before March 2020, please indicate the change in attendance for these two time periods:

	Increased	Slightly increased	Stayed the same	Slightly decreased	Decreased
March 2020-June 2021					
July 2021-December 2021					

15. Were there any workshops that you were not able to offer after March 2020? If so, please list them and provide a brief explanation.

16. Did you perceive a change in overall workshop satisfaction after March 2020?
    - Yes, increased satisfaction
    - Yes, slightly increased satisfaction
    - No change
    - Yes, slightly decreased satisfaction
    - Yes, decreased satisfaction

17. What was the basis of your perception?

18. If you taught online, what challenges did you and your colleagues experience? Please select all that apply.
    - Not applicable. I/we did not teach online.
    - Not being able to visually assess understanding
    - Online workshops often require additional support
    - Technology learning curve for instructors
    - Technology learning curve for participants

- Platform changes
- Hardware or software malfunctions
- Other, please specify below

19. If you taught online, what successes did you and your colleagues experience?
    - Not applicable. I/we did not teach online.
    - Different people could attend since they did not have to travel to a physical location
    - Increased participation
    - Increased interaction
    - Other, please specify below

20. In the future, our library will offer workshops…. Please select all that apply. We define hybrid as offering attendees the choice of attending the same live workshop in-person or online.
    - In-person
    - Online
    - Hybrid
    - Not applicable. We are discontinuing workshops.

21. Please include any other comments or observations regarding how the pandemic has affected workshops offerings, attendance, and future plans.

We thank you for your time spent taking this survey.
Your response has been recorded.
